# A Complete Analysis of HA and NA Genes of Influenza A Viruses

**DOI:** 10.1371/journal.pone.0014454

**Published:** 2010-12-29

**Authors:** Weifeng Shi, Fumin Lei, Chaodong Zhu, Fabian Sievers, Desmond G. Higgins

**Affiliations:** 1 The Conway Institute of Biomolecular and Biomedical Research, University College Dublin, Dublin, Ireland; 2 Institute of Zoology, Chinese Academy of Sciences, Beijing, China; University of Georgia, United States of America

## Abstract

**Background:**

More and more nucleotide sequences of type A influenza virus are available in public databases. Although these sequences have been the focus of many molecular epidemiological and phylogenetic analyses, most studies only deal with a few representative sequences. In this paper, we present a complete analysis of all Haemagglutinin (HA) and Neuraminidase (NA) gene sequences available to allow large scale analyses of the evolution and epidemiology of type A influenza.

**Methodology/Principal Findings:**

This paper describes an analysis and complete classification of all HA and NA gene sequences available in public databases using multivariate and phylogenetic methods.

**Conclusions/Significance:**

We analyzed 18975 HA sequences and divided them into 280 subgroups according to multivariate and phylogenetic analyses. Similarly, we divided 11362 NA sequences into 202 subgroups. Compared to previous analyses, this work is more detailed and comprehensive, especially for the bigger datasets. Therefore, it can be used to show the full and complex phylogenetic diversity and provides a framework for studying the molecular evolution and epidemiology of type A influenza virus. For more than 85% of type A influenza HA and NA sequences into GenBank, they are categorized in one unambiguous and unique group. Therefore, our results are a kind of genetic and phylogenetic annotation for influenza HA and NA sequences. In addition, sequences of swine influenza viruses come from 56 HA and 45 NA subgroups. Most of these subgroups also include viruses from other hosts indicating cross species transmission of the viruses between pigs and other hosts. Furthermore, the phylogenetic diversity of swine influenza viruses from Eurasia is greater than that of North American strains and both of them are becoming more diverse. Apart from viruses from human, pigs, birds and horses, viruses from other species show very low phylogenetic diversity. This might indicate that viruses have not become established in these species. Based on current evidence, there is no simple pattern of inter-hemisphere transmission of avian influenza viruses and it appears to happen sporadically. However, for H6 subtype avian influenza viruses, such transmissions might have happened very frequently and multiple and bidirectional transmission events might exist.

## Introduction

Influenza A virus is one of most important pathogens that infect humans, mammals, birds and poultry. The viral genomes are composed of eight separate segments encoding at least 11 proteins. Haemagglutinin (HA), encoded by the fourth segment, is an important glycoprotein and a major surface antigen which is responsible for attaching the virions to hosts, deciding the pathogenicity and virulence [1]. The sixth segment encodes another glycoprotein, neuraminidase (NA), which is a second major surface antigen associated with releasing newly produced viral particles, and drug resistance [1]. So far, 16 HA and 9 NA subtypes of type A influenza virus have been identified [2] and more than one hundred of the possible 144 HA-NA combinations have been found [3].

Some attempts have been made to analyze the phylogenetic diversity and distribution of type A influenza viruses [4]–[7]. Among them, Liu and colleagues published a “panorama phylogenetic analysis” of all the 16 HA and 9 NA subtypes and they divided them into 68 HA and 49 NA lineages and sublineages [6]. This study provided a comprehensive framework for studying the evolutionary and epidemiological history of type A influenza virus. However, this, like all previous analyses, just selected a small number of representative strains available for their phylogenetic analyses. In fact, they analyzed 1264 HA sequences and 1154 NA sequences. This limited sampling of sequences could make the results less conclusive and underestimate the real phylogenetic diversity of type A influenza especially for human H1N1 and H3N2 influenza viruses. In addition, the nomenclature system that they proposed was still ambiguous and less effective in that for some sequences it was hard find a lineage to which they belonged [5], [6].

The Influenza Virus Resource is a database of influenza sequences and associated information [8]. As of 31^th^ October, 2009, there were 22291 HA sequences and 13345 NA sequences of type A influenza viruses available. However, apart from subtype information, there is no other phylogenetic or genetic information available for the sequences. In particular, many sequences are not analyzed by anyone or some of them have been analyzed but there are no corresponding references in PubMed.

Pigs are regarded as the main intermediate host for avian influenza viruses to make the appropriate genetic changes in order to infect humans. They have both cell-receptors to match human and avian influenza viruses [9], [10] and several reports have provided genetic evidence to support this view [11], [12]. Influenza viruses of H1N1, H1N2 and H3N2 subtypes circulate widely in pig populations. Besides these, viruses of other subtypes, such as H3N1, H4N6, H5N1, H5N2 and H9N2 have been also reported to infect pigs [13]. However, the phylogenetic diversity of all swine influenza viruses (SIV) is not clear to date.

Apart from viruses from humans, birds and pigs, viruses have been isolated from other species, such as horses, dogs and mink. For example, highly pathogenic H5N1 avian influenza viruses have been isolated from tiger [14], leopard [14], cat [15], [16], dog [17], [18] and pika [19]. Although a few cases of infections caused by these extra species have been reported, phylogenetic diversity of these viruses has been seldom studied as a whole.

Migratory waterfowl of the world are natural reservoirs of avian influenza viruses (AIV) of all known subtypes. It has been long clear that AIV evolved into two separate lineages, the Eurasian and North American clades, due to geographic isolation [1] and there is limited virus exchange between them [20], [21]. Nonetheless, several inter-hemisphere transmission cases of AIV have been reported [22]–[24]. In particular, Olsen et al. proposed the global patterns of occurrence of influenza A virus in wild birds [25]. In addition, migratory birds are often regarded as being responsible for the wide and fast spread of highly pathogenic avian influenza (HPAI) H5N1 viruses in Eurasia and Africa [26]–[28], although this is still controversial [29]–[31]. Therefore, a complete analysis of inter-hemisphere transmission of AIV based on a large number of sequences is needed. To shed light on the above questions, a systematic and complete analysis using all HA and NA sequences available is needed. However, when the numbers of sequences exceed a few hundred, it becomes difficult to visualize and analyze a phylogenetic tree, which is a standard device to study the molecular evolution and epidemiology of viral outbreaks.

Principal Coordinates Analysis (PCOORD) [32] has been used by us in the past [33] and the accompanying software has been used to analyze virus sequence variation [34]. PCOORD takes a matrix of Euclidean distances between a set of objects and return a set of principal axes that try to preserve the distances. Multidimensional Scaling (MDS) methods work by finding a set of axes that minimize the “stress” between the original distance matrix and the distances between the plotted sequences [35]. If the distances are Euclidean, then this is known as classical MDS and is equivalent to PCOORD. The main difference is in the details of how the axes are calculated. MDS has also been used to visualize antigenic variation in influenza viruses [36] and there are fast algorithms and software available for applying this to very large data sets [37].

The main reason for not using PCOORD or MDS is the superior detail that can be seen by close inspection of phylogentic trees and the desire for cladistic classification schemes. The main reason in favor of using these methods is the ability to analyze and visualize data sets of more or less any size. A further advantage is the ability to visualize relationships where sequences which are intermediate between others are found such as happens if divergent virus sequences undergo recombination. Overall, we believe that a combination of methods is probably most useful where PCOORD is used to find the main groupings and to look for outliers and intermediates and where detailed groupings are either confirmed or discovered by phylogenetic analysis of subsets of sequences.

In this paper, we compiled the largest datasets of HA and NA genes of influenza and analyzed 85% of all HA and NA gene sequences available in GenBank by combining PCOORD and traditional phylogenetic trees. This work provides a framework for studying the molecular evolution and epidemiology of type A influenza and sheds light on the phylogenetic diversity of SIV and inter-hemisphere transmission of AIV.

## Materials and Methods

Nucleotide sequences were downloaded from the Influenza Virus Resource at the National Center for Biotechnology Information (NCBI) (http://www.ncbi.nlm.nih.gov/genomes/FLU/FLU.html) by using each of the 16 HA and 9 NA subtypes as search queries on October 31^st^, 2009. This gave 16 HA datasets and 9 NA datasets. Each dataset was aligned by Muscle [38] first and further adjusted manually in Bioedit [39]. The mature HA protein has two subunits, HA1 and HA2, connected by disulfide linkages [1]. HA1 has typically about 324 amino acids, while HA2 has about 222 amino acids. For sequences of H1 to H9 and H15, many of them were not full length and only HA1s were available. In order to include these sequences in the analysis, we removed the HA2 section from the alignment for those sequences which were full length. For the rest of the HA subtypes (H10 to H14, and H16), both HA1 and HA2 were used. In addition, sequences with more than 10 leading and/or 10 terminal gaps and lower quality sequences with more than 10 ambiguous nucleotide bases in each alignment were excluded from later analysis. This left, 18975 out of 22291 HA sequences and 11345 out of 13352 NA sequences to be analyzed. This is more than 85% of all the sequences that were available, at that time. Summary details of the HA and NA sequences from each subtype are given in Tables 1 and 2.

**Table 1:** Summary information and geographical origin for all HA sequences.

Subtypes	Africa	Asia	Europe	North America	Oceania	South America	Number of sequences available	Number of sequences into analysis	Length of alignment (bp)
H1	83	1110	911	2630	297	190	5221	4245	978
H2	2	59	42	169	7	7	286	258	1017
H3	142	3012	1918	3398	1145	460	10075	8662	969
H4	1	60	28	311	16		416	378	987
H5	406	2275	373	260	5		3319	2866	963
H6	4	182	40	262	7		495	464	987
H7	10	58	246	511	17	13	855	816	945
H8		2	4	42			48	37	1053
H9	2	1106	30	45	1		1184	907	960
H10	4	21	10	106			141	125	1581
H11	1	11	14	81	4		111	100	1596
H12		1	4	51	2		58	46	1623
H13		1	28	19		1	49	41	1587
H14		1	3				4	4	1649
H15					10		10	9	975
H16		1	13	5			19	17	1698
Total	655	7900	3664	7890	1511	671	22291	18975	

**Table 2:** Summary information and geographical origin for all NA sequences.

Subtypes	Africa	Asia	Europe	North America	Oceania	South America	Number of sequences available	Number of sequences into analysis	Length of alignment (bp)
N1	234	2344	1104	2194	223	30	6129	4932	1350
N2	19	1905	675	2293	662	53	5607	5034	1221
N3	2	76	68	160	7	9	322	275	1404
N4		6	7	44	6		63	57	1377
N5		16	4	71	13		104	87	1341
N6	1	72	24	249	4		350	254	1380
N7	5	17	32	116	4	4	178	153	1269
N8	6	55	44	328	24	7	464	451	1320
N9		27	4	87	9	1	128	119	1335
Total	267	4518	1962	5542	952	104	13345	11362	

A distance matrix for every dataset was calculated using Kimura's two-parameter model [40]. This was performed using DNADIST in Phylip 3.68 [41]. These distance matrices were first analyzed to produce “ordinations”. These ordinations embed N nucleotide sequences as vectors into an M-dimensional space, where M≪N, such that the distances of the end-points of the vectors resemble the “true” distances d_ij_ obtained by DNADIST.

There are different approaches to determining these vectors. Often the ordination is formulated as an optimization problem, which in our case can be stated analytically in terms of a matrix eigen decomposition. Firstly, we transform the distance matrix into an association matrix with elements a_ij_ = −(1/2)d_ij_^2^. This association matrix is then centered by subtracting its row- and column-means and adding the overall mean. The final task is to find the eigenvectors of this centered matrix. As we are looking for a low-dimensional embedding (typically just 2- or 3-dimensional), we only have to find the eigenvectors corresponding to the M eigenvalues of the greatest magnitude.

Eigenvectors corresponding to the largest eigenvalues point in the directions of the greatest variability of the data. If the distances are sufficiently well behaved (a close approximation to Euclidean distances), then these M eigenvalues will be positive. If not all but only the leading eigenvectors are sought, one could employ a power-iteration. Convergence of the power method is geometric in the ratio of the first two eigenvalues. If these eigenvalues are similar in size, then convergence of the power method will be slow.

Calculating all the distances of N sequences has a time complexity of O(N^2^) while performing the singular value decomposition (SVD) has a time complexity of O(N^3^). In our study the largest N was 8662 and scalability is not yet an issue. For this work, therefore, we used the Python standard implementation of SVD from the NUMPY library and its dependencies. Our program for PCOORD is available on request from the authors. Alternatively, the SPACER program [33], written in Fortran and performing PCCORD, is available on-line from http://www.hiv.lanl.gov/content/sequence/PCOORD/PCOORD.html. However, for problems where N>10,000, alternative algorithms may become necessary. Such methods could be Split-and-Combine MDS (SCMDS) [37] or the Nyström method [42].

In the last step of PCCORD, we visualized the data in two dimensions by simply plotting the first versus the second or the first versus the third axes, with greatest associated eigenvalues. In each PCCORD figure, each dot represents for one sequence. The color of the dot signifies where the virus was isolated from and the shape indicates the host.

Next, phylogenetic trees were estimated using the same datasets. For datasets with more than 2500 sequences, Neighbor-Joining (NJ) trees [43] were constructed using the linux version of PAUP* 4b10 [44]. The distance model was set to HKY85 [45] and all other parameters were set to default. For datasets with 1500 to 2500 sequences, phylogenetic trees were estimated using the Maximum Likelihood (ML) method as implemented in PhyML [46]. The general time-reversible (GTR) model [47] was applied as the model of nucleotide substitution and base frequency was estimated by maximizing the likelihood of the phylogeny. In addition, the proportion of the invariable sites was estimated rather than fixed and four substitution rate categories were used with the gamma distribution parameter estimated to account for variable substitution rates among sites. All other parameters were set to default. For datasets of less than 1500 sequences, ML trees were also estimated in PhyML using the same parameters but with 100 bootstrap replicates. All the trees were visualized using Dendroscope [48]. It should be noted that for most of trees, we did not specify a sequence as an outgroup. However, for some trees whose clusters were not very clear, we selected a sequence from a neighboring group as an outgroup to root these trees.

**Figure 1:**
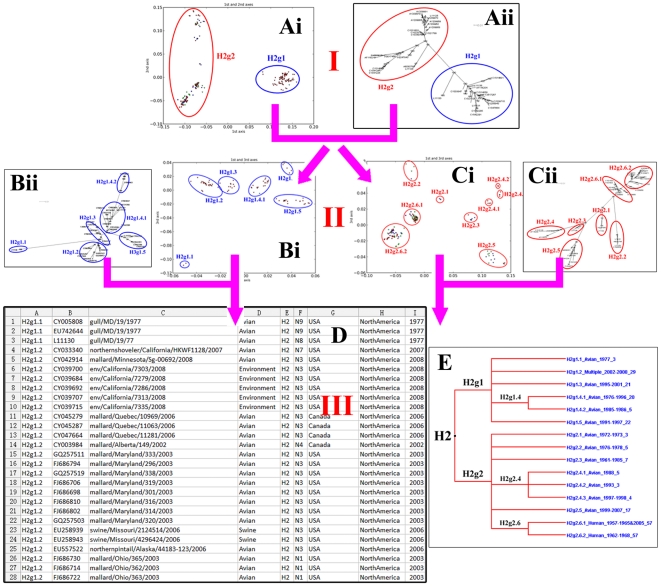
The workflow. This figure takes HA sequences of H2 subtype as an example to illustrate the workflow. In step I, we carry out a PCOORD (Ai) and a phylogenetic analysis (Aii) using the H2HA dataset. Results from the two methods support their division into two groups, H2g1 and H2g2. In step II, we repeat step I using two sub-datasets, H2g1 (Bi and Bii) and H2g2 (Ci and Cii). Bi and Bii show the results from PCOORD and the phylogenetic tree using H2g1. Group H2g1 is further divided into 5 subgroups. Similarly, Ci and Cii display the results from PCOORD and the phylogenetic tree using H2g2, and this group can also be further divided into some smaller subgroups. In step III, we summarize the results into a table (D) and in a tree-like figure (E). Panel E summarizes the phylogenetic diversity of HA sequences of H2 influenza A virus and values after the underlines indicate the numbers of sequences in the group or subgroup. If there are groups can be further divided based on the results of step II, we will repeat step II until there are no distinctly separated groups or the bootstrap values are too low to support further sub-division.

The remainder of the analysis consisted of using the ordinations and trees to identify subgroups of sequences, with clear separation from the rest. The aim was to identify groups of clearly related sequences which we could use to ask questions about epidemiology. This was mainly done by visual inspection of PCOORD results and by using the bootstrap values from the trees. Sometimes, host and geography information was also used to help to define the groups. In most cases, the groups seen in the ordinations and trees agreed. When they disagreed, the trees were used. This process was repeated iteratively by re-applying PCCORD and phylogenetic analysis to smaller and smaller groups of sequences until the groups showed no clear subgroups and/or bootstrap values for subgroups became too low (Figure 1). The result was a small hierarchy of groupings for each HA and NA subtype. The subtypes were divided into groups and subgroups. The groups were named according to the subtype using the notation: H*n*g*m* where *n* was the HA subtype and *m* was the subgroup of HA sequences. Similarly NA sequences were named using the same notation N*n*g*m*. These groups might be subdivided into subgroups using a period “.” and a further integere.g. H2g2.3 is subgroup 3 of group 2 of subtype 2 of the HA sequences. This was similar to the notation system of Liu et al [6]. All the PCCORD figures, phylogenetic trees and other supporting materials were available from http://myosin.ucd.ie/~shiwf/influenzaclassification/index.html.

We summarized all the groupings in two large tables, one for HA (Table S1) and one for NA (Table S2). These are available from the website: http://myosin.ucd.ie/~shiwf/influenzaclassification/index.html. These tables form the basis of a database with one record for each sequence which stores basic information about each sequence, including its grouping. Table S1 has 16 sections which correspond to 16 HA subtypes, while Table S2 has 9. Each entry records 9 pieces of information. The first column gives the grouping from this analysis. The second column gives the GenBank accession number of the sequence. Columns 3 to 9 give the sequence name, virus host, viral HA and NA subtype, country of isolation, continent and time of isolation. Therefore, for each sequence in the present analysis, if you search using its GenBank accession number, you can find a clear and unambiguous group to which it belongs. In addition, a tree-like figure was drawn to show the simple hierarchy of the groupings for each HA and NA subtype. An example is shown in Figure 1E. The 23 trees for the 23 subtypes are available from http://myosin.ucd.ie/~shiwf/influenzaclassification/index.html.

## Results

### Phylogenetic diversity of HA and NA genes

In this paper we take 22291 HA sequences and 13345 NA sequences from GenBank. After removing short and low quality sequences, we analyze 18975 HA and 11362 NA sequences (Tables 1 and 2). By far the majority of HA sequences belong to subtypes H1, H3 H5, and H9 which account for ∼88% of the total. Approximately 88% of NA sequences belong to N1 and N2. In addition, most sequences are isolated from viruses from North America and Asia, and only a few are isolated from Africa and South America.

H14 and H15 are only represented by 4 and 9 sequences respectively and are not subdivided. All of the subtypes were divided into a total of 280 HA subgroups (Table S1) and 202 NA subgroups (Table S2). Many subgroups only include a few sequences and subgroups composed of one to five sequences account for 32.5% and 36.1% of all HA and NA subgroups, respectively.

**Figure 2:**
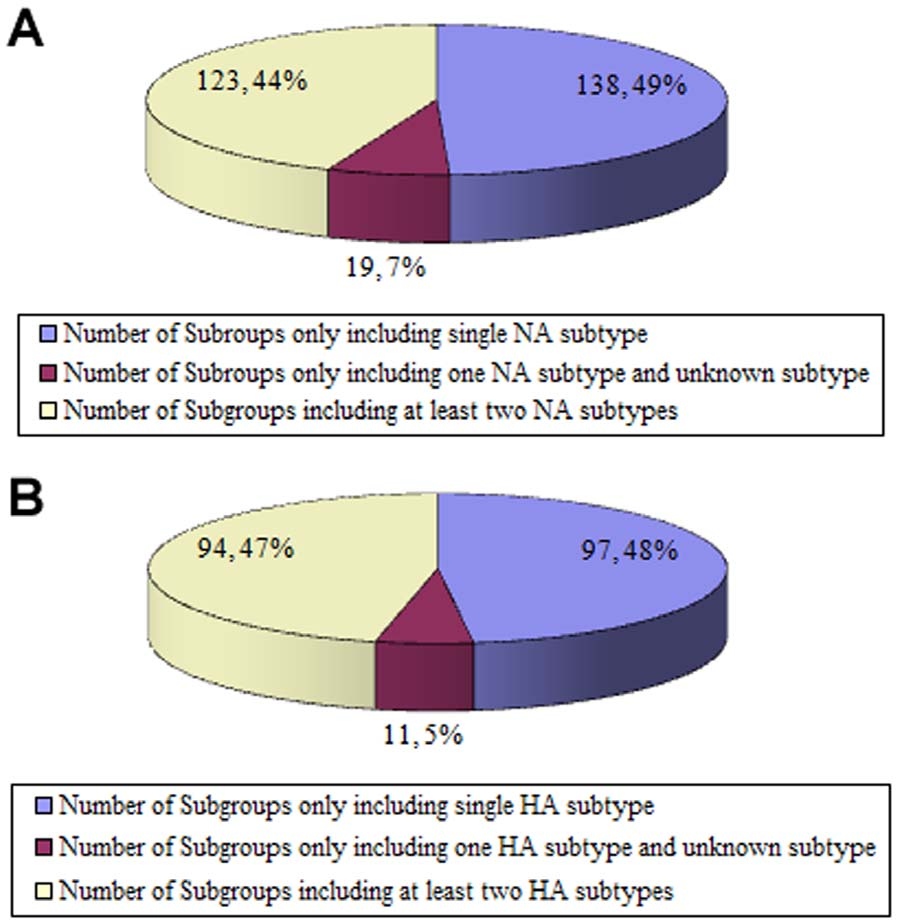
Phylogenetic diversity indicated by the association of HA and NA subtypes. Panel A shows the phylogenetic diversity indicated by the NA subtype distribution among HA subgroups, while panel B shows the phylogenetic diversity indicated by the HA subtype distribution among NA subgroups.

We analyze the association of HA and NA subtypes in isolates in different HA and NA subgroups (Figure 2). 140 HA subgroups only include isolates associated with a single NA subtype, while 123 HA subgroups include sequences of at least two NA subtypes (Figure 2A). Similarly, 97 NA subgroups only include sequences associated with a single HA subtype, while 94 NA subgroups include sequences of at least two HA subtypes (Figure 2B).

Generally, most of the results of the classification are consistent with a recent analysis which attempted an overall classification of flu sequences [6]. However, this work is more detailed and comprehensive, especially for the bigger datasets, such as for subtypes H1, H3, H5, H9, N1 and N2.

### Phylogenetic diversity of the main subtypes

**Figure 3:**
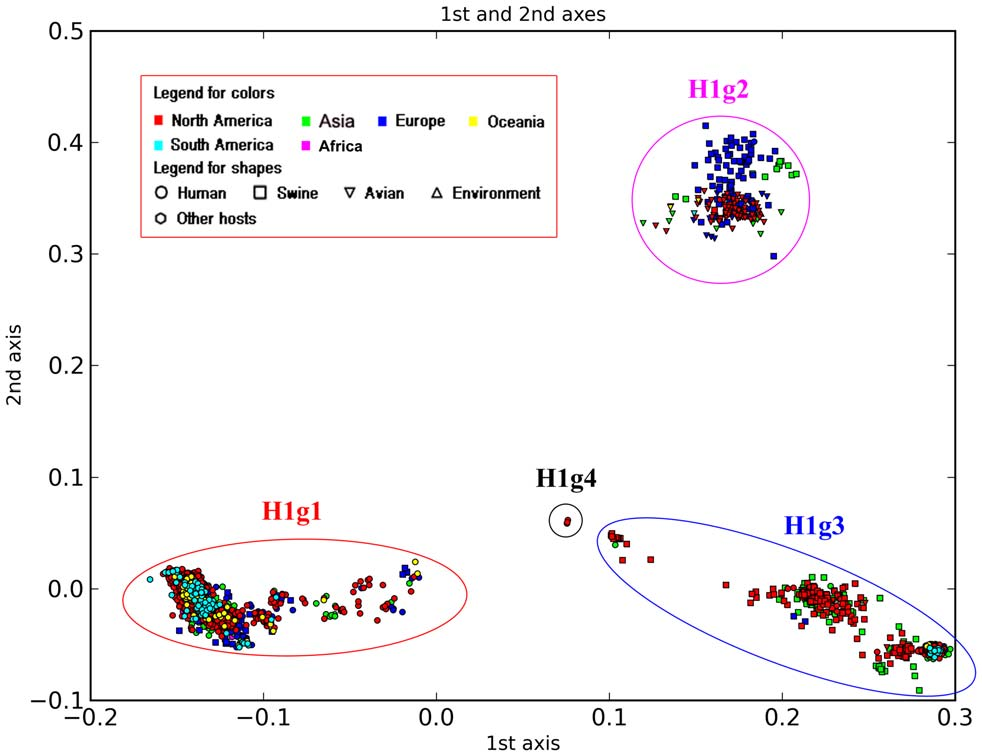
PCCORD of H1 subtype influenza viruses. In this figure, each sequence is shown as a dot using shape to signify host and color to indicate geographic region (the continent this virus was isolated from). For Figure 4 to [Fig pone-0014454-g005] [Fig pone-0014454-g006] [Fig pone-0014454-g007] 8 and all PCCORD figures available from our webpage, we use the same shape and color coding.

In detail, H1 is divided into 64 subgroups (Table 3). First, it is divided into four major groups labeled H1g1 to H1g4 (Figure 3). H1g1 is the seasonal human H1N1 influenza group, which largely corresponds to h1.2 in Liu et al [6]. Apart from the 1918 sequences, Liu et al. further divided this lineage into three sublineages, h1.2.2, h1.2.3 and h1.2.5. However, we further divide this group into five subgroups, largely corresponding to the periods 1933∼1957, 1948∼1984, 1986∼2001, 1994∼2008 and 2004∼2009, respectively. In addition, these five subgroups have been further subdivided in our scheme. In particular, swine influenza viruses of H1N2 subtype isolated from Europe (H1g1.3) also fall within this group, and this is consistent with h1.2.4 reported by Liu et al [6]. H1g2 is composed of sequences from Eurasian pigs and worldwide birds. We further subdivide H1g2 into two subgroups rather than the three sublineages made by Liu et al [6]. In detail, H1g2.1, consisting of nine smaller subgroups, is composed of virus sequences from North American birds. H1g2.2 is composed of seven subgroups, with H1g2.2.1 to H1g2.2.3 including viruses from Eurasian birds and H1g2.2.4 to H1g2.2.7 including viruses from Eurasian pigs. This is better to clarify the avian origin of Eurasian swine influenza [49], [50]. H1g3 includes classical swine sequences (H1g3.1 to H1g3.3) and pandemic H1N1 human influenza (H1g1.4). Liu et al. subdivided the classical swine lineage into two sublineages, h1.3.1 and h1.3.2. However, we believe that viruses of h1.3.2 have diversified since the late 1990s and should be divided into two subgroups, H1g3.2 and H1g3.3. In particular, viruses of H1g3.3 are the most likely progenitors of the pandemic 2009 H1N1 virus HA genes [12]. Liu et al. classified the 1918 sequences within the seasonal human H1N1 lineage as h1.2.1 [6]. However, due to the discovery that HA of seasonal human H1N1 is not derived from Spanish flu directly [51], we define the 1918 sequence as an independent group, H1g4.

**Table 3:** Number of subgroups defined in this analysis.

HA		NA	
HA subtype	Number of subgroups	NA subtype	Number of subgroups
H1	64	N1	64
H2	15	N2	48
H3	38	N3	25
H4	19	N4	8
H5	41	N5	3
H6	17	N6	13
H7	22	N7	16
H8	9	N8	16
H9	25	N9	9
H10	2		
H11	12		
H12	6		
H13	4		
H14	1		
H15	1		
H16	4		

**Figure 4:**
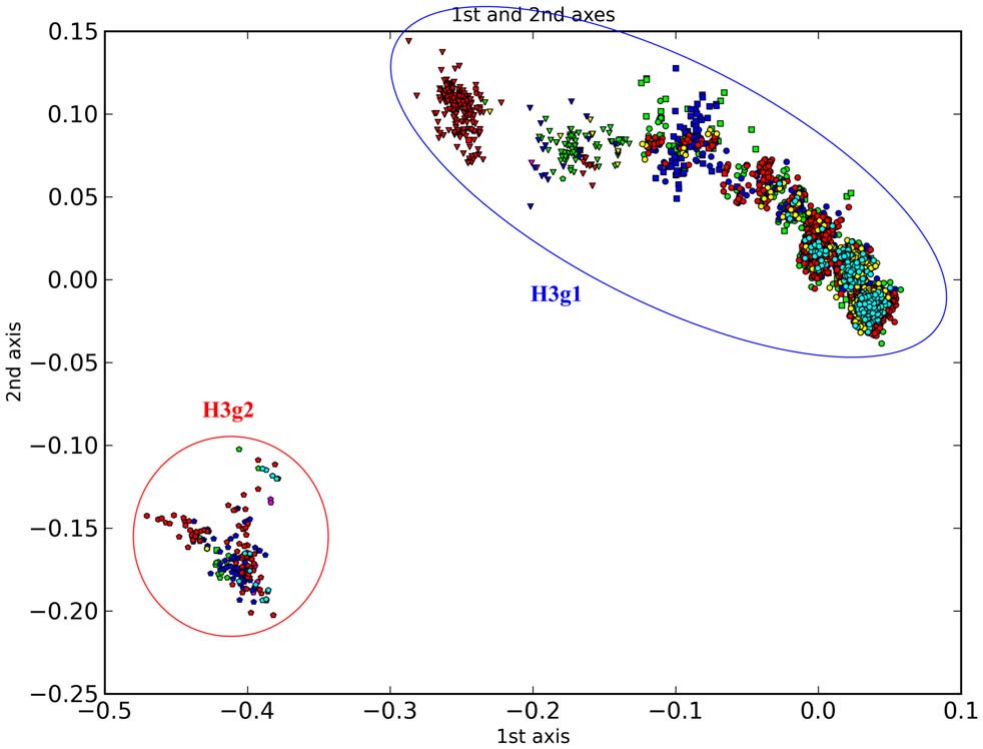
PCCORD of H3 subtype influenza viruses. The sequences are coded for host (shape of dot) and geographic origin (color) as for figure 3.

H3 includes two major groups which are, in turn, subdivided into 38 subgroups (Figure 4, Table 3). H3g1 is mainly composed of worldwide avian H3 sequences and human and swine H3N2 influenza sequences, while H3g2 is an H3N8 equine group. Liu et al. divided H3 into three lineages [6]. For human and swine H3N2 influenza, thousands of HA sequences were deposited in GenBank. Liu et al. just classified them into four sublineages, h3.1.3 to h3.1.6. However, we classify them into 13 subgroups H3g1.4 to H3g1.16 along with smaller subdivisions. Meanwhile, for h3.2 which was defined by Liu et al. with two sublineages, we classify them into seven subgroups, largely corresponding to the periods 1971∼1972 (H3g2.1), 1963∼1969 (H3g2.2), 1976∼1984 (H3g2.3), 1985∼1987 (H3g2.4), 1989∼2007 (H3g2.5), 1986∼2006 (H3g2.6) and 1999∼2008 (H3g2.7). The canine influenza viruses fall within H3g2.7 [52]. Apart from H3g1 and H3g2 which we define here, Liu et al. defined a third lineage which they named as h3.3 and further subdivided it into h3.3.1 and h3.3.2. However, h3.3.1 only contains one short sequence and is excluded from our analysis. In addition, h3.3.2 also contains just one sequence and we define it to be within H3g1.13 rather than a single lineage outside the two main groups and this is consistent with a recent report [53].

**Figure 5 pone-0014454-g005:**
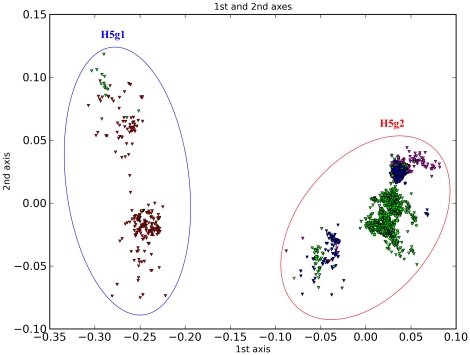
PCCORD of H5 subtype influenza viruses. The sequences are coded for host (shape of dot) and geographic origin (color) as for figure 3.

H5 is mostly composed of avian influenza sequences and can be divided into two main groups (Figure 5). H5g1 is mostly composed of sequences from North America with a small number from Asia, while H5g2 is mainly composed of sequences from Eurasia and Africa and most of them are H5N1 subtype. Liu et al. defined A/Turkey/Ramon/73 (H5N2) as a third group (h5.3). In our scheme, we classify it within H5g2.1 as an independent subgroup, H5g2.1.1. Overall, H5 is divided into 41 subgroups (Table 3). The highly pathogenic avian H5N1 influenza viruses fall within 16 subgroups (Table S1). Compared to the nomenclature system proposed by WHO [54], some of our subgroups are less detailed. Nonetheless, many clades numbered by WHO correspond to the groupings reported here (Table S3). In detail, clades 0, 1, 3–6, 8 and 9 fall within H5g2.2.2, and clade 7 falls within H5g2.2.1 and H5g2.2.2. In particular, clades 2.2, 2.3.1, 2.3.2, 2.3.3 and 2.3.4 correspond to our groupings perfectly. In addition, due to the inclusion of new sequences recently deposited in GenBank, we further report six subgroups that are not included into the WHO nomenclature system (Table S3).

**Figure 6 pone-0014454-g006:**
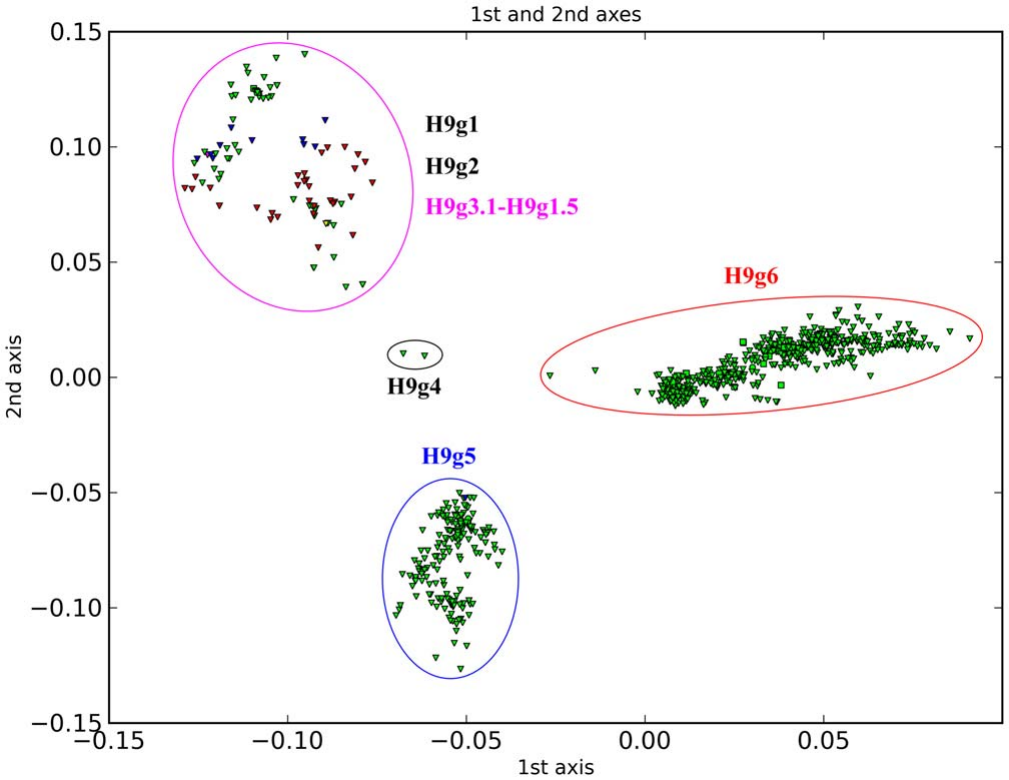
PCCORD of H9 subtype influenza viruses. The sequences are coded for host (shape of dot) and geographic origin (color) as for figure 3.

H9 is divided into six main groups (Figure 6). Although the groups defined here are numbered differently to the lineages defined by Liu et al., the arrangements mostly correspond closely to each other [6]. Our H9g1, H9g2 and H9g3 correspond to h9.1, h9.2 and h9.3 defined by Liu et al [6]. In detail, H9g1 includes H9N2 avian influenza sequences from North America from 1966 and from China from 1998 and 2000. H9g2 is composed of avian H9N2 influenza sequences from the 1990s. H9g3 includes avian sequences of multiple subtypes from both hemispheres. In addition, H9g5 corresponds to h9.4.1, and H9g4 and H9g6 correspond to h9.4.2. These three groups are composed of H9N2 sequences from Asia from different time points. However, based on the main groups, we further subdivide them into 25 subgroups (Table 3).

**Figure 7 pone-0014454-g007:**
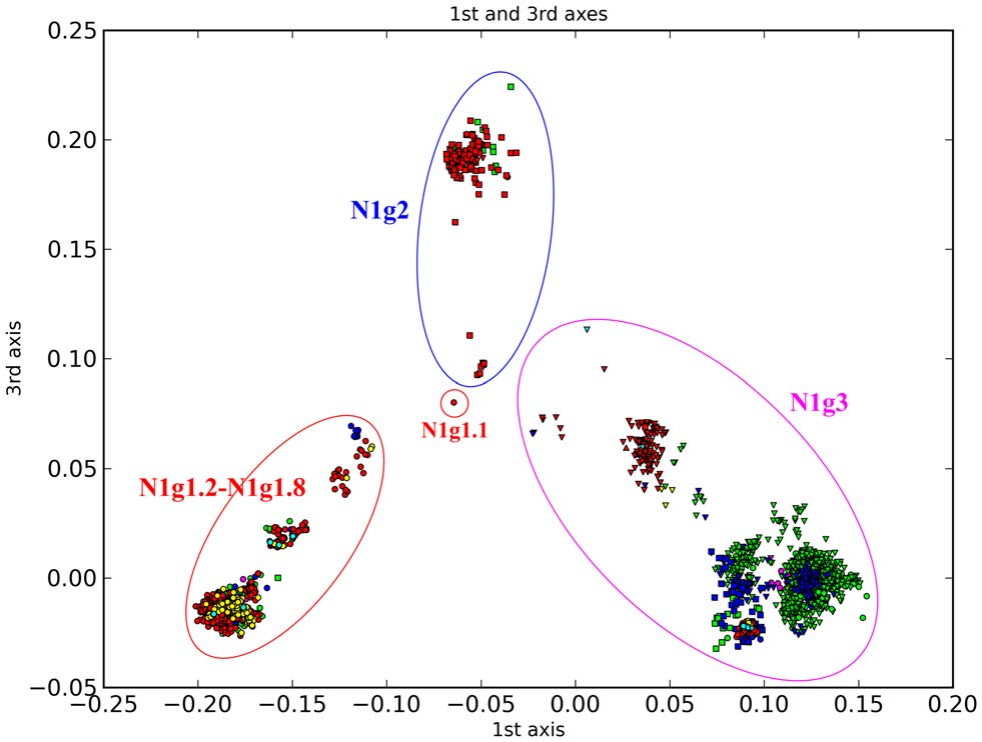
PCCORD of N1 subtype influenza viruses. The sequences are coded for host (shape of dot) and geographic origin (color) as for figure 3.

Similar to Liu et al. and a previous report [4], N1 is divided into three big groups (Figure 7) [6]. N1g1 is the seasonal human H1N1 group. Liu et al. defined this lineage (n1.2) into four sublineages. However, we further classify this group into eight subgroups (N1g1.1 to N1g1.8) and some of these subgroups have been further subdivided, such as N1g1.2 and N1g1.5 to N1g1.8. N1g2, which corresponds to n1.3 in Liu et al., is the classical swine group and includes four subgroups with distinct temporal features. N1g3 is composed of sequences from Eurasian pigs and worldwide avian hosts. Here we differ from the previous arrangement [6] in that we firstly divide this group into two subgroups. N1g3.1 mostly includes viruses from birds, while N1g3.2 includes viruses from Eurasian pigs (N1g3.2.1) and the pandemic human H1N1 (N1g3.2.2). Most of the highly pathogenic avian H5N1 influenza viruses fall within N1g3.1.13, while a few fall within N1g3.1.12. In total, N1 is subdivided into 64 subgroups (Table 3).

**Figure 8:**
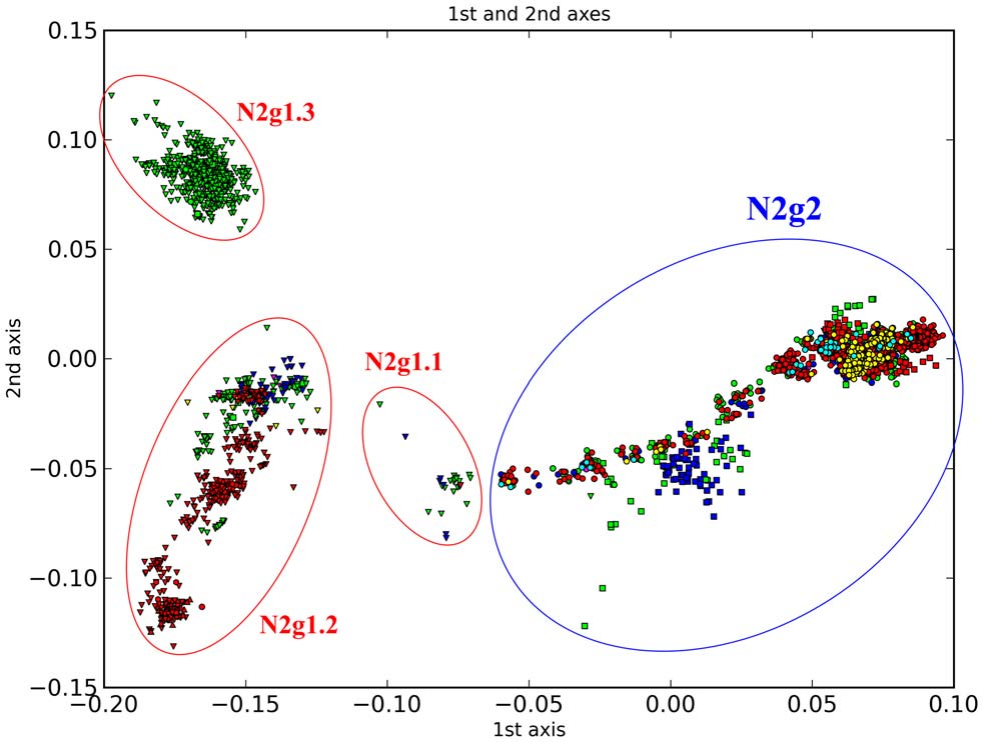
PCCORD of N2 subtype influenza viruses. The sequences are coded for host (shape of dot) and geographic origin (color) as for figure 3.

N2 can be divided into two groups, mainly based on PCCORD and host information (Figure 8), and this is consistent with Liu et al [6]. N2g1 is composed of avian sequences from all over the world. The associated HA subtypes mainly include H9, H5 and H6. N2g2 is mostly composed of sequences of human and swine H3N2 influenza viruses. However, our further groupings are different from those of Liu et al. They subdivided the avian group into four sublineages and the mammalian group into seven sublineages. In contrast, we divide the avian group (N2g1) into three subgroups (N2g1.1 to N2g1.3) and the mammalian group (N2g2) into 20 subgroups (N2g2.1 to N2g2.20) each with characteristic temporal and host features. In total, all N2 sequences are classified into 48 subgroups (Table 3).

### Phylogenetic diversity of the minor subtypes

Consistent with Liu et al. [6], H2 is divided into two groups (Figure S1). H2g1 is mainly composed of avian influenza sequences from North America and is further divided into five subgroups, H2g1.1 to H2g1.5. H2g2 consists of both worldwide avian influenza sequences (H2g2.1 to H2g2.5) and worldwide human influenza sequences of H2N2 subtype (H2g2.6). However, Liu et al. also reported a North American sublineage, h2.2.4 and they reported the rest of the sublineages as coming from the Eastern hemisphere. In contrast to this result, we find some viruses from North America fall within three subgroups, H2g2.3 to H2g2.5, with Eurasian strains rather than forming a separate sublineage.

H13 is divided into three groups (Figure S2). H13g1 is composed of avian influenza sequences of N6 and N9 subtypes from Europe and the Western hemisphere. H13g2 consists of avian influenza sequences of N2 and N6 subtypes from North America, while H13g3 includes avian influenza sequences of multiple subtypes from Eurasia and North America. This is consistent with the arrangement made by Liu et al. However, we further subdivide H13g3 into two subgroups. H13g3.1 is from Eurasia from 1979 to 2002 and viruses in this subgroup are from N2, N4 and N6 subtypes. However, viruses in H13g3.2 are isolated from North America from 2004 to 2008 and all of them belong to the N9 subtype.

All H16 viruses are isolated from birds and of H16N3 subtype. Liu et al. did not analyze this subtype due to there being too few sequences available. However, the 17 sequences analyzed can be clearly divided into two groups (Figure S3). H16g1 includes sequences from Europe from 1983 and 1999. In addition, we further divide H16g2 into three subgroups. H16g2.1 includes viruses from North America from 1975 to 1998, while H16g2.2 is composed of viruses from Eurasia from 1976 to 1999. H16g2.3 is more complicated and viruses in this subgroup come from both Europe and North America from 2006.

Similar to the arrangement made by Liu et al., N3 viruses are divided into two groups (Figure S4). N3g1 is further divided into three subgroups that are largely from North America, Eurasia and South America, respectively, while N3g2 includes a few sequences from Europe and North America from 1975 to 2006. Likewise, N9 is also divided into three groups and they are mainly from North America, Eurasia and South America, respectively, although some Eurasian strains fall with the North American group (Figure S5). Liu et al. did not include the South American virus; however, we define it as an independent group (N9g3) to emphasize its special phylogenetic relationship with those from Eurasia and North America.

Subtypes H7 (Figure S6), N7 and N8 are all divided into three groups and this is consistent with Liu et al. The first group is mostly composed of avian influenza sequences from the Western hemisphere. The second group mostly consists of avian influenza sequences from the Eastern hemisphere and the third group mainly includes sequences from worldwide horses. These three subtypes are further subdivided into 22, 16 and 16 subgroups, respectively. In particularly, highly pathogenic H7N7 viruses that have caused human infections in the Netherlands [55] fall within H7g2.3.7 and N7g2.1. Moreover, highly pathogenic H7N3 viruses that caused human infections in Canada in 2004 [55] fall within H7g1.1.4.3 and N3g1.1.10.

The rest of the subtypes, including H4 (Figure S7), H6, H8, H10, H11, H12, N4, N5 and N6, are largely composed of avian influenza sequences and viruses of these subtypes have evolved into two groups, the Eastern hemisphere group and the Western hemisphere group. These groupings are consistent with the arrangement by Liu et al [6].

### Phylogenetic diversity of Swine Influenza Viruses (SIV)

**Figure 9:**
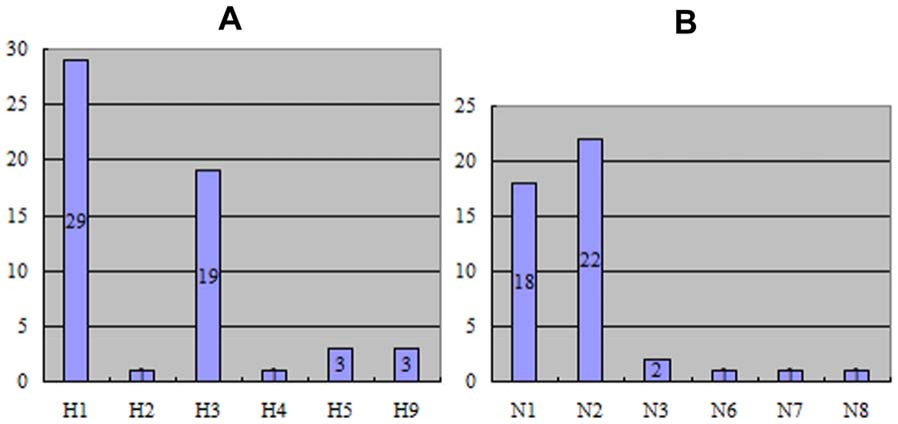
Numbers of subgroups associated with SIV. Panel A shows the numbers of subgroups associated with swine influenza viruses among different HA subtypes, while panel B shows the numbers of subgroups among different NA subtypes.

Our datasets include 717 HA sequences (∼3.78% of the total) and 519 NA sequences (∼4.57% of the total) isolated from pigs. Apart from some sequences whose HA or NA subtypes are unknown, the HA subtypes of the rest of the sequences are mainly H1-H5 and H9 (Figure 9). The NA subtypes are N1-N3 and N6-N8. In addition, there are a total of 12 HA and NA combinations identified (Table 4). In particular, swine HA sequences all come from 56 of the 280 HA subgroups, while swine NA sequences all come from 45 of the 202 NA subgroups (Figure 9).

**Table 4:** HA phylogenetic diversity of SIV.

HA subtype	NA subtype*	Number of groups	Lists of the groups
H1	N0	5	H1g1.1.2,H1g3.2.1,H1g3.2.2,H1g3.2.3,H1g3.3
	N1	19	H1g1.1.2,H1g1.3.2,H1g1.3.5.2.2,H1g1.4.1.1,H1g1.4.1.3, H1g1.5.3,H1g2.1.9.1,H1g2.2.2,H1g2.2.4,H1g2.2.5,H1g2.2.6, H1g2.2.7,H1g3.1,H1g3.2.1,H1g3.2.2,H1g3.2.3,H1g3.2.4, H1g3.3,H1g3.4
	N2	18	H1g1.3.1,H1g1.3.3,H1g1.3.4,H1g1.3.5.1,H1g1.3.5.2.1, H1g1.3.5.2.2,H1g1.3.5.3.1,H1g1.3.5.3.2,H1g1.5.1,H1g1.5.2, H1g1.5.3,H1g2.2.6,H1g2.2.7,H1g3.2.1,H1g3.2.2,H1g3.2.3, H1g3.2.4,H1g3.3
	N7	1	H1g1.2.1.1
H2	N3	1	H2g1.2
H3	N0	1	H3g1.6.3
	N1	3	H3g1.6.3, H3g1.8, H3g1.13
	N2	17	H3g1.3.1,H3g1.3.2,H3g1.4,H3g1.5,H3g1.6.1,H3g1.6.2, H3g1.6.3, H3g1.7,H3g1.8,H3g1.9,H3g1.10,H3g1.11, H3g1.12, H3g1.13,H3g1.14.1,H3g1.14.2,H3g1.15.2
	N3	1	H3g1.1.2.3
	N8	1	H3g2.5
H4	N6	1	H4g1.4.2
H5	N1	2	H5g2.2.2, H5g2.2.3.2
	N2	1	H5g2.1.6
H9	N2	3	H9g3.5, H9g6.2, H9g6.3.2

*N0 means NA subtype is unknown.

From a host point of view, among the 56 HA subgroups, only 15 Eurasian subgroups and two North American subgroups are composed entirely of sequences from pigs (Table 5). Among these, 9 subgroups belonging to H1g1.3 fall within H1g1, which is the main seasonal human H1N1 group. The remaining 39 subgroups contain viruses from pigs and one or two other main host types. 11 subgroups include viruses from pigs and avian hosts and 17 subgroups include viruses from pigs and humans (Table 5). In particular, in 10 subgroups, viruses from pigs, avian hosts and humans cluster together (Table 5). It should also be noted, SIV cluster with equine influenza viruses in one subgroup (Table 5).

**Table 5:** Phylogenetic relationship between SIV and viruses from other hosts†.

	Eurasia	North America	Both
Only swine influenza	H1g1.3.1, H1g1.3.2, H1g1.3.3, H1g1.3.4, H1g1.3.5.1, H1g1.3.5.2.1, H1g1.3.5.2.2, H1g1.3.5.3.1, H1g1.3.5.3.2, H1g2.2.4, H1g2.2.6, H3g1.5, H3g1.6.1, H3g1.6.2, H3g1.8(15*)	H1g1.5.1, H1g1.5.2(2)	
With avian influenza	H1g2.2.2, H1g2.2.5, H3g1.3.1, H3g1.3.2, H5g2.1.6, H9g3.5, H9g6.2(7)	H1g2.1.9.1, H2g1.2, H3g1.1.2.3, H4g1.4.2(4)	
With human influenza	H1g1.1.2, H1g1.2.1.1, H1g1.4.1.1, H1g1.4.1.3, H1g2.2.7, H1g3.2.4, H3g1.6.3, H3g1.9, H3g1.10, H3g1.11, H3g1.12, H3g1.14.1, H3g1.15.2(13)	H1g3.1, H3g1.7(2)	H3g1.4, H3g1.14.2(2)
With avian and human influenza	H5g2.2.2, H5g2.2.3.2, H9g6.3.2(3)	H1g1.5.3, H1g3.4(2)	H1g3.2.1, H1g3.2.2, H1g3.2.3, H1g3.3, H3g1.13(5)
With equine influenza	H3g2.5(1)		

† In this table, Eurasia means all SIV in the subgroups listed in the column are all isolated from Eurasia. Similarly, North America means all SIV in the subgroups listed in the column are isolated from North America. Both means the SIV in the subgroups listed in the column are isolated from Eurasia and North America.

*Numbers in the parentheses indicate the number of subgroups in this cell.

Further analysis of the 56 SIV HA subgroups indicate that the phylogenetic diversity of Eurasian SIV is greater than that of North American SIV and both of them are getting more and more complex over time (Table 6). This is caused not only by the increased phylogenetic complexity of H1N1, H1N2 and H3N2, but also by the involvement of more subtypes, such as H3N8, H5N1 and H9N2 in Eurasia and H4N6 in North America (Table 6).

**Table 6:** Temporal change of phylogenetic diversity of SIV.

	Eurasia	North America
1930s-1940s	H1N1(1)*	H1N1(1)
1950s		H1N1(1)
1960s	H3N2(1)	H1N1(1)
1970s	H1N1(2), H3N2(2)	H1N1(1), H3N2(1)
1980s	H1N1(3), H1N2(1), H3N2(6)	H1N1(2), H3N2(1)
1990s	H1N1(8), H1N2(6), H1N7(1) H3N2(9), H9N2(2),	H1N1(2), H1N2(1) H3N2(3), H4N6(1)
2000s	H1N1(9), H1N2(11), H3N1(3), H3N2(10), H3N8(1), H5N1(2), H5N2(1), H9N2(3)	H1N1(6), H1N2(6), H2N3(1), H3N1(1), H3N2(1), H3N3(1)

*Numbers in the parentheses indicate the number of subgroups in this cell.

### Phylogenetic diversity of viruses from species other than human, pigs or birds

Apart from the three main host types of birds, pigs and humans, a small number of influenza viruses have also been isolated from other species. In this analysis, there are only 280 HA sequences and 137 NA sequences isolated from these extra species. Both HA and NA data show that apart from equine influenza viruses, viruses from the rest of the species come from a very small number of subgroups (Table 7). Equine influenza viruses fall within 11 HA subgroups and five NA subgroups. In detail, viruses of the H3N8 subtype from all over the world come from 10 HA subgroups and four NA subgroups, while those of the H7N7 subtype come from one HA subgroup and one NA subgroup.

**Table 7:** Phylogenetic diversity of influenza viruses from species other than human, pigs or birds.

Virus host	Number of subgroups	
	HA	NA
Blow fly	1	1
Dog	2	2
Cat	3	1
Civet	1	1
Horse	11	5
Ferret	1	*
Giant anteater	1	1
Leopard	1	*
Mink	3	3
Musk rat	1	1
Pika	3	1
Raccoon dog	1	1
Seal	3	1
Stone marten	1	1
Tiger	2	1
Whale	1	1

*no sequence data available.

### Inter-hemisphere transmission of AIV

Based on the groupings, we identify potential inter-hemisphere transmission events of AIV (Tables 8) based on the following rules. First of all, AIV evolved into two separate lineages, the Eurasian and North American clades, due to geographic isolation [1]. In practice, we determine whether a subgroup comes from a Eurasian lineage or a North American lineage based on the PCCORD and phylogenetic analysis. If a subgroup is defined as a Eurasian lineage, occurrence of any virus coming from North America and falling within this subgroup is regarded as a potential inter-hemisphere transmission event and vice versa. Secondly, if there is no convincing evidence to define which lineage one subgroup comes from, then we use timing to help to judge the transmission direction. Generally, the oldest virus in a subgroup is regarded as the prototype virus and correspondingly the transmission direction can be inferred. Thirdly, if there are only a few sequences available and we cannot make an unambiguous decision regarding transmission direction, the transmission direction is regarded as unclear. If it has been reported before, but we do not agree with that report, the transmission direction is also regarded as unclear. If the results got by the first and the second rule are not consistent, we believe that the transmission direction is not clear.

**Table 8:** Subgroups associated with inter-hemisphere transmission of AIV.

Transmission direction	Subgroups	
	HA	NA
Eastern hemisphere to Western hemisphere	H3g1.3.2 [56], H5g2.1.1 [57], H5g2.1.6, H7g2.2, H9g3.1 [23], H9g3.2 [23], H9g3.3, H13g3.2	N6g2.3, N6g2.4, N9g2
Western hemisphere to Eastern hemisphere	H3g1.1.3.2, H4g1.5.2 [58], H5g1.4.3.1, H5g1.4.3.2, H5g1.5.1.1 [59], H5g1.5.4 [60], H6g1.1, H7g1.1.2, H8g2.1, H9g1 [61], H11g1.2.3, H13g1	N1g3.1.5.1, N1g3.1.5.2.1, N1g3.1.5.2.8, N2g1.2.1.1 [60], N2g1.2.2 [59], N6g1.3, N6g1.5, N8g1.1.1, N8g1.1.2, N8g1.1.3.2, N8g1.1.3.4, N9g1.2, N9g1.3
Multiple and bidirectional transmission	H6g2.3 [23]	
Transmission direction is not clear.	H2g2.3, H2g2.1.4.1, H2g2.1.4.2, H2g2.1.4.3, H2g2.5, H16 [20]	N2g1.1, N2g1.2.4.2, N2g1.2.4.3, N3g2, N7g2.2.1

Both the HA and NA data in Table 8 show some examples of unidirectional inter-hemisphere transmission of AIV that appears to have occurred in some subgroups. The associated viruses come from subtypes H2-H9, H11, H13, H16, N1-N3 and N6-N9. In subgroups H3g1.3.2, H5g2.1.1, H5g2.1.4, H7g2.2, H9g3.1, H9g3.2, H9g3.3, H13g3.2, N6g2.3, N6g2.4 and N9g2, some viruses are estimated to be transmitted from the Eastern hemisphere to the Western hemisphere. In contrast, some AIV appear to have been transmitted from the Western hemisphere to the Eastern hemisphere and the associated viruses fall within several subgroups, most clearly in subgroups H4g1.5.2, H5g1.5.1.1 and N2g1.2.2. However, in some subgroups, such as H2g2.3, H2g2.1.4.1, H2g2.5 and N3g2, the transmission direction is not clear.

Notably, in subgroup H6g2.3, multiple and bidirectional transmission of AIV might have happened. On the whole, H6g2 is a Eurasian lineage [23]. In H6g2.3.1 it appears that H6N2 virus might be have been transmitted from North America to Europe, but H6g2.3.2 suggests the opposite transmission direction, from Asia to North America. In H6g2.3.3.1 we also see a possible inter-hemisphere transmission from North America to Europe, although one virus (pintail/Alberta/179/93) in this subgroup has been believed to belong to the Eurasian lineage [23]. That subgroup H6g2.3.3.2 is entirely composed of sequences from North American birds from 1998 to 2005 and might indicate these Eurasia-like viruses have become established in North American birds after being transmitted. In H6g2.3.3.3, some sequences from strains from North American birds from 2005 to 2007 cluster together with those from European avian strains from 1998 to 2007. This might suggest another cross hemisphere transmission event from Europe to North America. Similarly, in H6g2.3.4.3, some strains from North American birds from 2004 to 2008 cluster together with those from Asian avian strains from 1997 to 2006. This also indicates that these North American avian strains might have been transmitted from Asia. Therefore, for H6 subtype AIV, multiple and bidirectional transmission between the two hemispheres might have occurred and some viruses might have become established in the new hemisphere after being transmitted.

## Discussion

In this paper, we compile a comprehensive dataset and carry out a PCCORD and phylogenetic analysis of HA and NA genes of type A influenza. Each of the 18975 HA sequences and 11362 NA sequences, is classified into an unambiguous and unique group. This can be used as an addition to the annotation of influenza HA and NA sequences deposited in GenBank. Our work provides new information for sequences, many of which have no associated literature. This also provides a clear framework for the study of the evolution and epidemiology of type A influenza and will help to answer some important influenza questions.

Compared to previous work, our results are more detailed and complete, especially for the bigger datasets of H1, H3, H5, H9, N1, and N2 subtypes that have thousands of sequences available. Among them, H1N1 and H3N2 influenza viruses are the two subtypes that seasonally circulate and infect humans, while H1N1, H1N2 and H3N2 are the important SIV subtypes. Further, H5N1 and H9N2 are the two AIV subtypes that have been widely circulating since late 1990s and have the ability to infect mammals including humans. Therefore, this work provides a complete analysis of viruses isolated from humans, pigs and avian hosts, which are the three main host types of type A influenza virus.

On the one hand, 138 out of 280 HA subgroups and 123 out of 202 NA subgroups include viral sequences of at least two subtypes. On the other hand, 32.5% of the HA and 36.1% of the NA subgroups are composed of no more than 5 sequences. Both of them lead to a challenge in selecting representative sequences in phylogenetic analyses. Undoubtedly, any analysis with inappropriate sampling will underestimate the phylogenetic diversities of type A influenza.

It should be noted that new sequences are deposited in GenBank on a daily basis. We just analyzed the sequences available at one time point. However, these groups can be combined with sequence or profile search software, such as BLAST [62] to allow the automatic classification of new sequences. Our results can also be used to help select suitable representative sequences in order to analyze new virus sequences.

Although only a few sequences are isolated from birds from South America, most of them are not similar to avian strains from other continents and mainly form independent subgroups, such as H1g2.1.2, H7g1.2, H8g2.1, N3g1.3 and N9g3. This indicates that the phylogenetic diversity of avian strains from South America is unique. Moreover, limited sampling might have underestimated the phylogenetic diversity of AIV from South America. Also, there is evidence that AIV from South America may evolve independently [63]. Therefore, extensive surveillance in wild birds is needed to better understand the ecology and evolution of AIV in South America.

Although sequences isolated from pigs make up a small percentage of all sequences available, they come from more than 20% of all subgroups. As the three main subtypes that have established in swine populations, viruses of H1N1, H1N2 and H3N2 subtypes fall within 45 different subgroups and account for most variation of SIV. Viruses of other subtypes have also been sporadically detected in pigs, although whether these transiently detected viruses have established is not clear [13]. However, considering the fact that pigs can be infected with H1-H13 avian influenza viruses under experimental conditions [64] and the increased phylogenetic diversity of SIV, we think that there is an increasing risk of generation of novel influenza viruses in pigs [65].

Among the 56 SIV associated HA subgroups, 17 subgroups only include sequences from pigs. In fact, except for H1g2.2.4 and H1g2.2.6, the other 9 subgroups of H1 fall within H1g1, the seasonal human H1 group. Also, for the four H3 groups, they do not form separate lineages. Therefore, in most SIV subgroups identified here, SIV sequences cluster with sequences from avian and/or human strains suggesting frequent inter-species transmission of the viruses between pigs and other hosts. Once again, this supports the mixing vessel hypothesis that pigs can infect both avian and human influenza viruses and can generate pandemic reassortants to infect humans [66].

Generally, the phylogenetic diversity of Eurasian SIV is greater than that of North American SIV and both of them are becoming more diverse over time. Notably, in the 1990s and 2000s, there were viruses belonging to more than 20 different subgroups circulating in Eurasia. Together with both the appalling Spanish flu in the late 1910s and the pandemic H1N1 in 2009 associated with SIV [12], [51], there is clearly a great need to maintain surveillance of the circulation and evolution of SIV.

Equine influenza viruses of the H3N8 subtype have become well established in horse populations [67]. In this analysis, equine influenza viruses fall within 11 HA and 5 NA subgroups and have shown a certain amount of phylogenetic diversity. For viruses from the other species, they fall within just a few subgroups. This suggests that the phylogenetic diversity of these viruses is very low and they have not become established among these species. Most of these infections occur sporadically and are most likely caused by direct or indirect contact with infected birds [14]–[18], although tiger-to-tiger transmission might exist [68].

It is has been long known that AIV evolved into two separate lineages, the Eurasian and North American clades, due to geographic isolation and there is limited virus exchange between them [20], [21]. Although inter-hemisphere transmission of AIV does not appear to be very frequent, our results indicate that inter-hemisphere transmission of AIV might have happened in viruses of many HA and NA subtypes with no clear pattern. This is consistent with a global pattern of AIV in wild birds proposed by Olen *et al.* by taking into account the ecology of virus hosts [25]. In the H6 subtype in particular, multiple and bi-directional inter-hemisphere transmission is estimated to have happened frequently. Undoubtedly, this increases the genetic exchange between AIV from the two hemispheres and emphasizes the need to maintain surveillance of AIV.

There has been much debate whether the HPAI H5N1 virus can be transmitted to North America by migratory birds. Some believe that there is little possibility for such transmission [20], while in other cases, the possibility could not be excluded [69], [70]. However, our results also show some evidence of a virus transmission route from Asia or Europe to North America. Experimental studies show that there is an asymptomatic period for birds after HPAI H5N1 infection [71]–[72]. During this period, birds can fly over a long distance. Another recent report reveals that the possible spread routes of H5N1 AIV from November 2003 to December 2006 match the flyways of migratory birds [28]. Taken together, we believe that it is possible for HPAI H5N1 to be introduced into North America via wild bird migration.

For some groups, such as H5g2.2.1 and N1g3.1.13, we did not perform a detailed analysis due to the facts that sequences in these groups are highly similar and PCCORD is not suitable to display small sequence differences among them. Instead, these groups could be further analyzed, purely using conventional phylogenetic analysis.

However, the combination of PCOORD and phylogenetic analysis provides a powerful way to analyze large datasets. On the one hand, PCOORD can analyze large datasets very quickly to find the main groups. Based on PCOORD, large datasets can be further divided into several smaller datasets. In addition, by applying different coloring and coding strategies, it becomes more convenient to display the temporal, geographic and host information which makes it easier to find inter-continent, inter-hemisphere, and cross host transmission of the viruses. On the other hand, phylogenetic trees estimated using smaller datasets can get more detailed information and can display small differences between similar sequences. Although there are some programs available that can visualize huge trees with hundreds or thousands of sequences [73], it is still a daunting task to analyze such big trees.

Taken together, we analyzed more than 85% HA and NA sequences of type A influenza available in GenBank, using both PCOORD and phylogenetic analysis. Our results provide a framework for studying molecular evolution and epidemiology of type A influenza and are a kind of new annotation to these sequences based on sequence similarities and phylogenies. Based on the above, we have been able to study the phylogenetic diversity of SIV and shed light on the role of pigs in the inter-species transmission of influenza. In addition, we have also studied potential inter-hemisphere transmission of AIV and found that multiple and bidirectional transmission might exist in H6 subtype AIV.

## Supporting Information

Table S1 The HA subgroups identified in this analysis. This table includes all HA subgroups identified in this analysis.
(3.58 MB XLS)

Table S2 The NA subgroups identified in this analysis. This table includes all NA subgroups identified in this analysis.
(2.15 MB XLS)

Table S3 A comparison of the WHO H5N1 nomenclature to the groupings reported by us.
(0.04 MB DOC)

Figure S1 PCCORD of H2 subtype influenza viruses. The sequences are coded for host (shape of dot) and geographic origin (color) as for figure 3.
(1.47 MB TIF)

Figure S2 PCCORD of H13 subtype influenza viruses. The sequences are coded for host (shape of dot) and geographic origin (color) as for figure 3.
(1.26 MB TIF)

Figure S3 PCCORD of H16 subtype influenza viruses. The sequences are coded for host (shape of dot) and geographic origin (color) as for figure 3.
(1.31 MB TIF)

Figure S4 PCCORD of N3 subtype influenza viruses. The sequences are coded for host (shape of dot) and geographic origin (color) as for figure 3.
(1.36 MB TIF)

Figure S5 PCCORD of N9 subtype influenza viruses. The sequences are coded for host (shape of dot) and geographic origin (color) as for figure 3.
(1.45 MB TIF)

Figure S6 PCCORD of H7 subtype influenza viruses. The sequences are coded for host (shape of dot) and geographic origin (color) as for figure 3.
(1.47 MB TIF)

Figure S7 PCCORD of H4 subtype influenza viruses. The sequences are coded for host (shape of dot) and geographic origin (color) as for figure 3.
(1.60 MB TIF)
