# Supplementary material for: A Complete Analysis of HA and NA Genes of Influenza A Viruses
Source: PLoS One. 2010 Dec 29;5(12):e14454. doi: 10.1371/journal.pone.0014454 (PMC3012125; doi:10.1371/journal.pone.0014454)
Supplement: Table S3 — A comparison of the WHO H5N1 nomenclature to the groupings reported by us. (0.04 MB DOC) [file pone.0014454.s003.doc]

Table S3. A comparison of the groupings reported by us to WHO H5N1 nomenclature.

|  | WHO H5N1 nomenclature | Groupings reported by us |
| --- | --- | --- |
| 1 | 0 | H5g2.2.2 |
| 2 | 1 | H5g2.2.2 |
| 3 | 3 | H5g2.2.2 |
| 4 | 4 | H5g2.2.2 |
| 5 | 5 | H5g2.2.2 |
| 6 | 6 | H5g2.2.2 |
| 7 | 7 | H5g2.2.1, H5g2.2.2 |
| 8 | 8 | H5g2.2.2 |
| 9 | 9 | H5g2.2.2 |
| 10 | 10 | H5g2.2.2 |
| 11 | 2.1.1 | H5g2.2.3.2 |
| 12 | 2.1.2 | H5g2.2.3.2 |
| 13 | 2.1.3 | H5g2.2.3.3 |
| 14 | 2.4 | H5g2.2.3.2 |
| 15 | 2.5 | H5g2.2.3.1, H5g2.2.3.2 |
| 16 | 2.2 | H5g2.3.1 |
| 17 | 2.3.1 | H5g2.2.4.3 |
| 18 | 2.3.2 | H5g2.2.4.4 |
| 19 | 2.3.3 | H5g2.2.4.2 |
| 20 | 2.3.4 | H5g2.2.4.1 |
| 21 |  | H5g2.2.4.5, H5g2.2.4.6, H5g2.2.4.7, H5g2.2.4.8, H5g2.3.2, H5g2.3.3 |
