# Supplementary figures and images for: A Complete Analysis of HA and NA Genes of Influenza A Viruses
Source: PLoS One. 2010 Dec 29;5(12):e14454. doi: 10.1371/journal.pone.0014454 (PMC3012125; doi:10.1371/journal.pone.0014454)

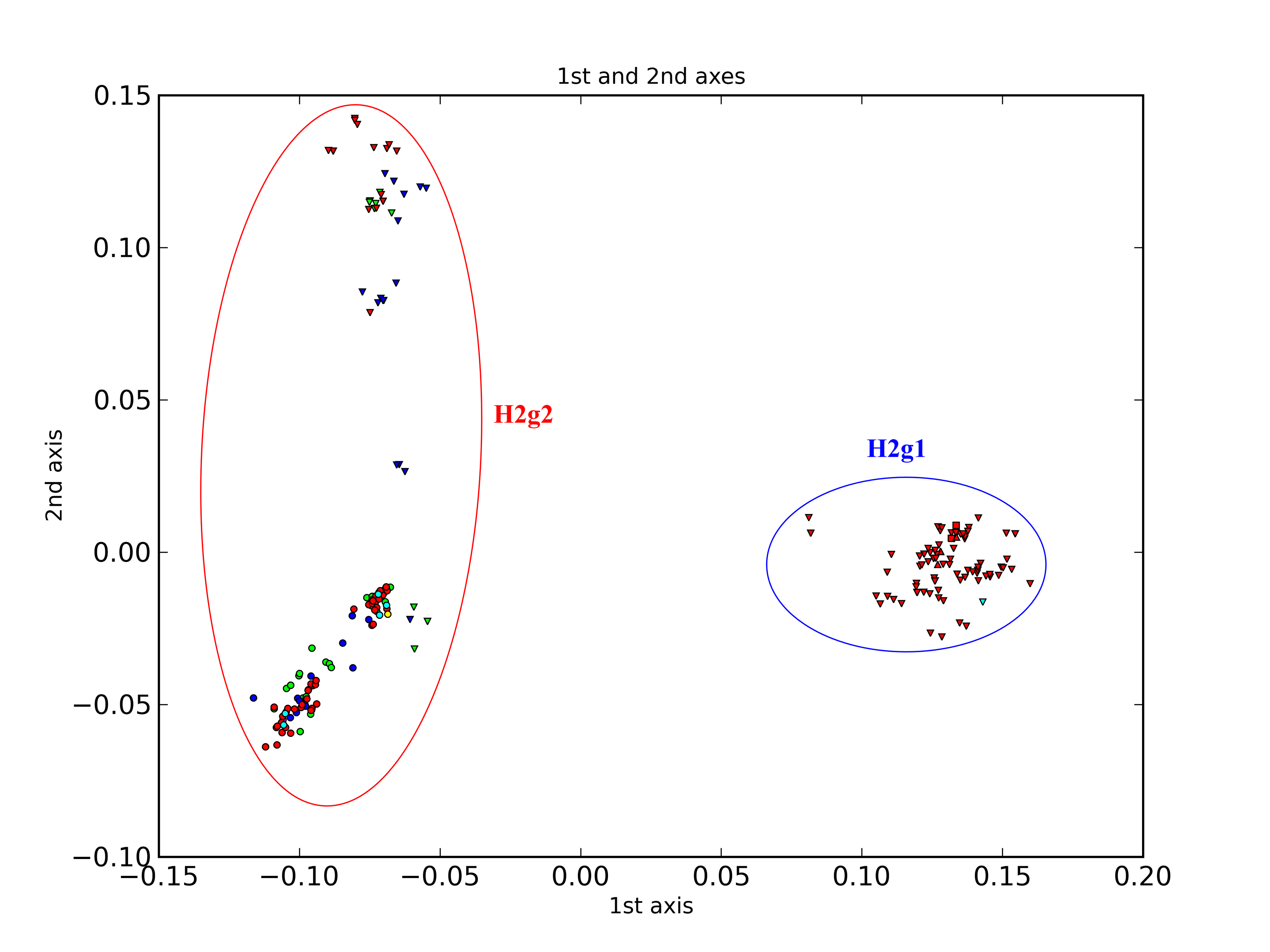

Supplement: Figure S1 — PCCORD of H2 subtype influenza viruses. The sequences are coded for host (shape of dot) and geographic origin (color) as for figure 3. (1.47 MB TIF) [file pone.0014454.s004.tif]

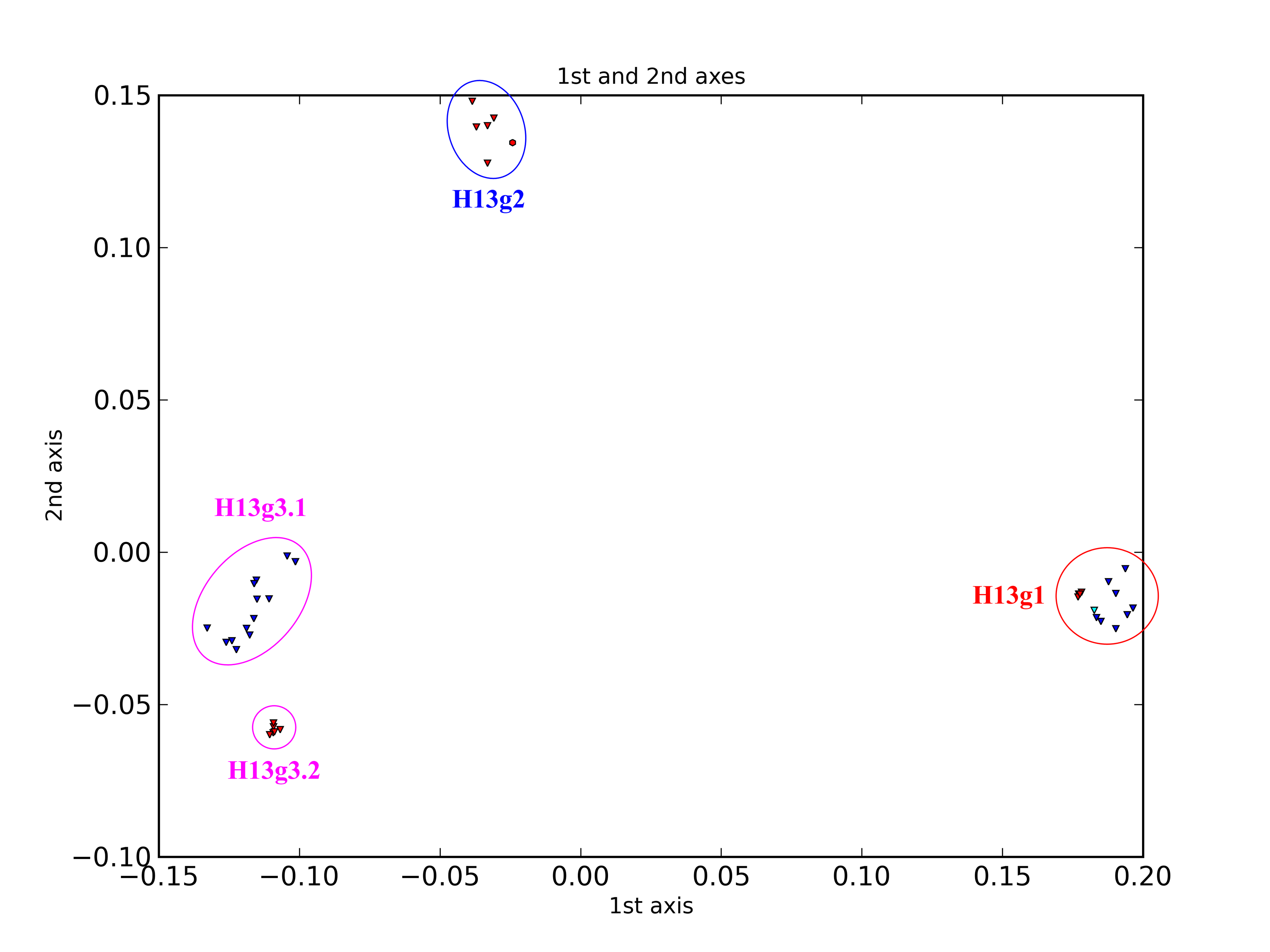

Supplement: Figure S2 — PCCORD of H13 subtype influenza viruses. The sequences are coded for host (shape of dot) and geographic origin (color) as for figure 3. (1.26 MB TIF) [file pone.0014454.s005.tif]

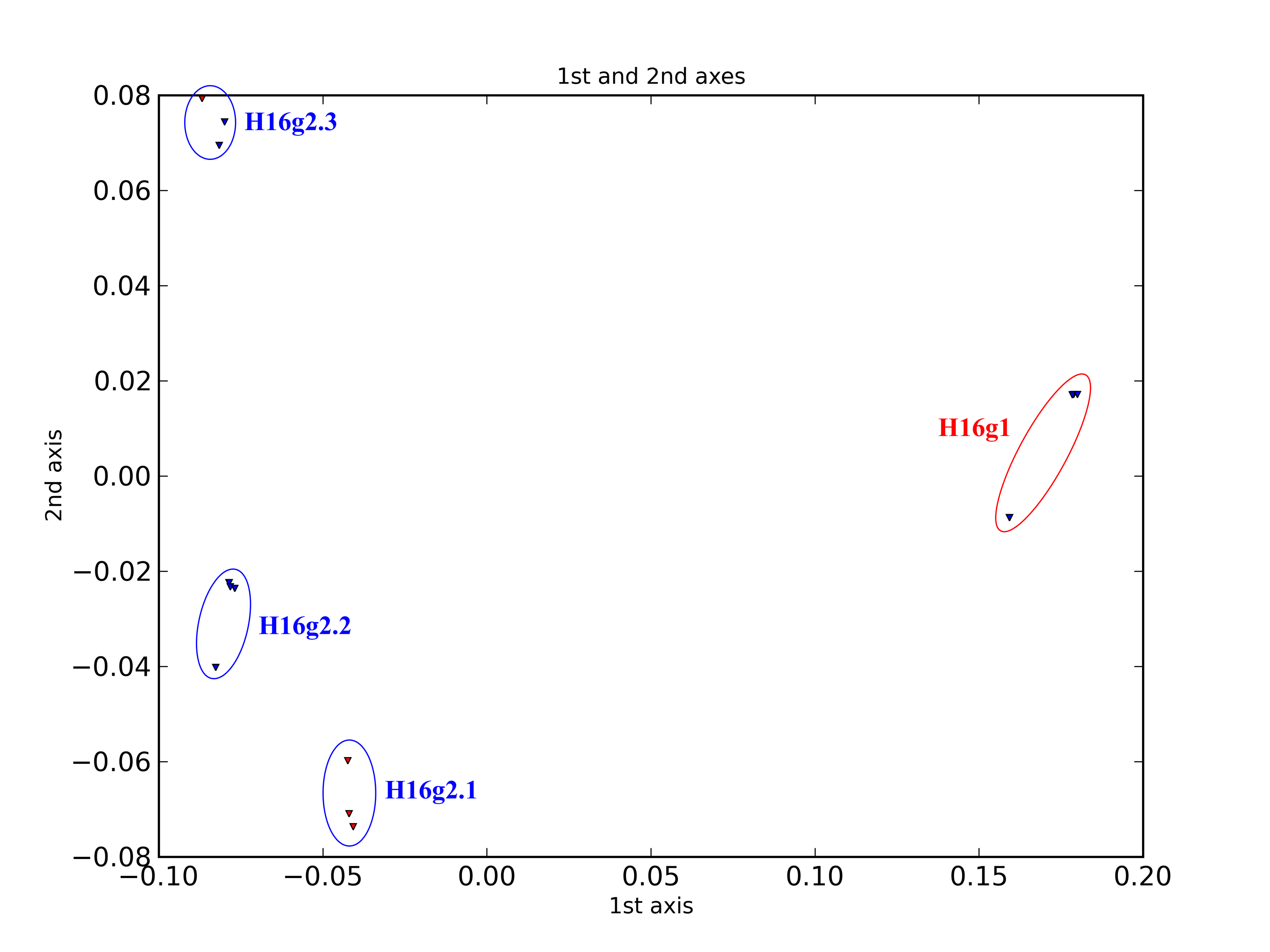

Supplement: Figure S3 — PCCORD of H16 subtype influenza viruses. The sequences are coded for host (shape of dot) and geographic origin (color) as for figure 3. (1.31 MB TIF) [file pone.0014454.s006.tif]

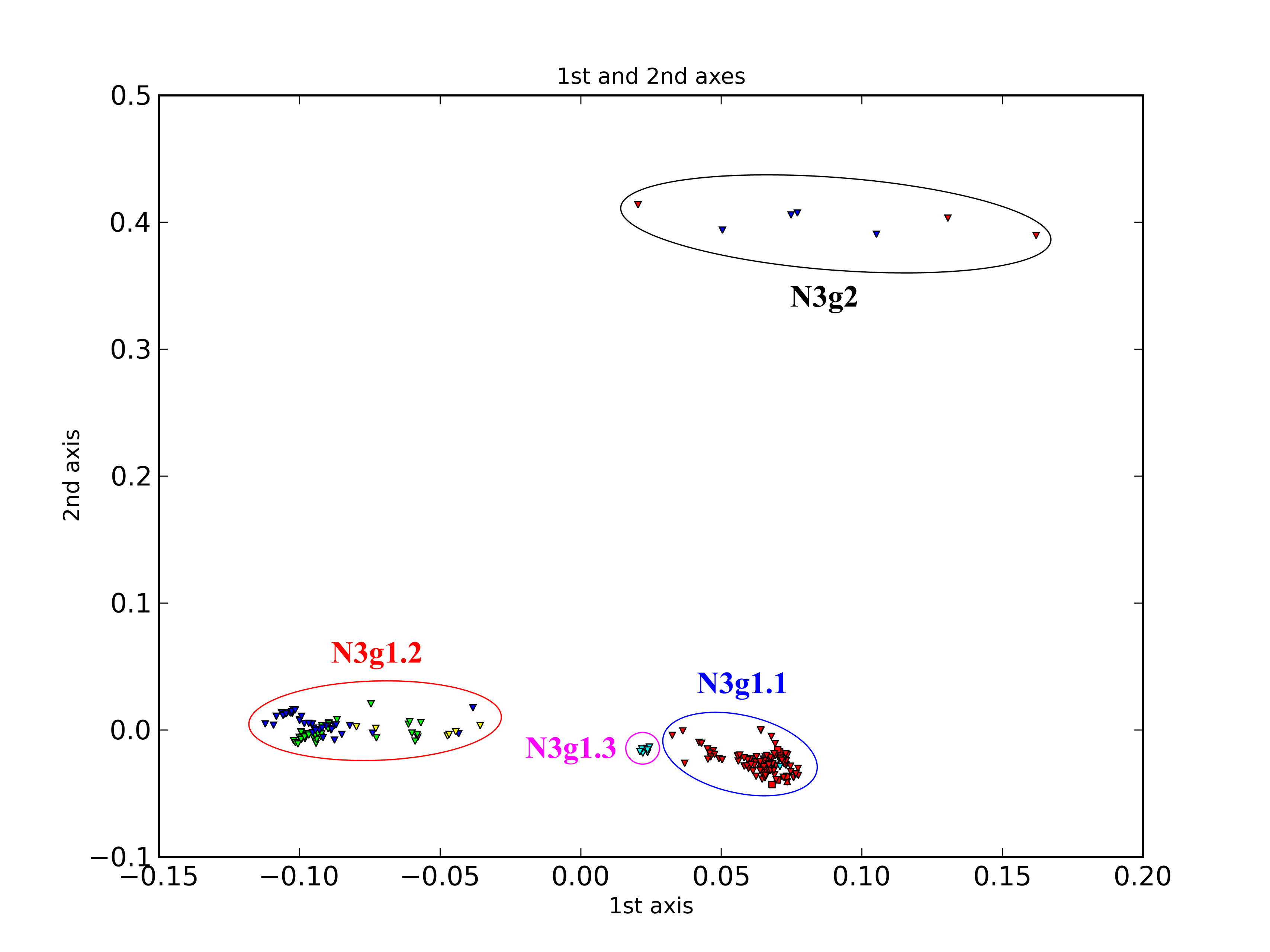

Supplement: Figure S4 — PCCORD of N3 subtype influenza viruses. The sequences are coded for host (shape of dot) and geographic origin (color) as for figure 3. (1.36 MB TIF) [file pone.0014454.s007.tif]

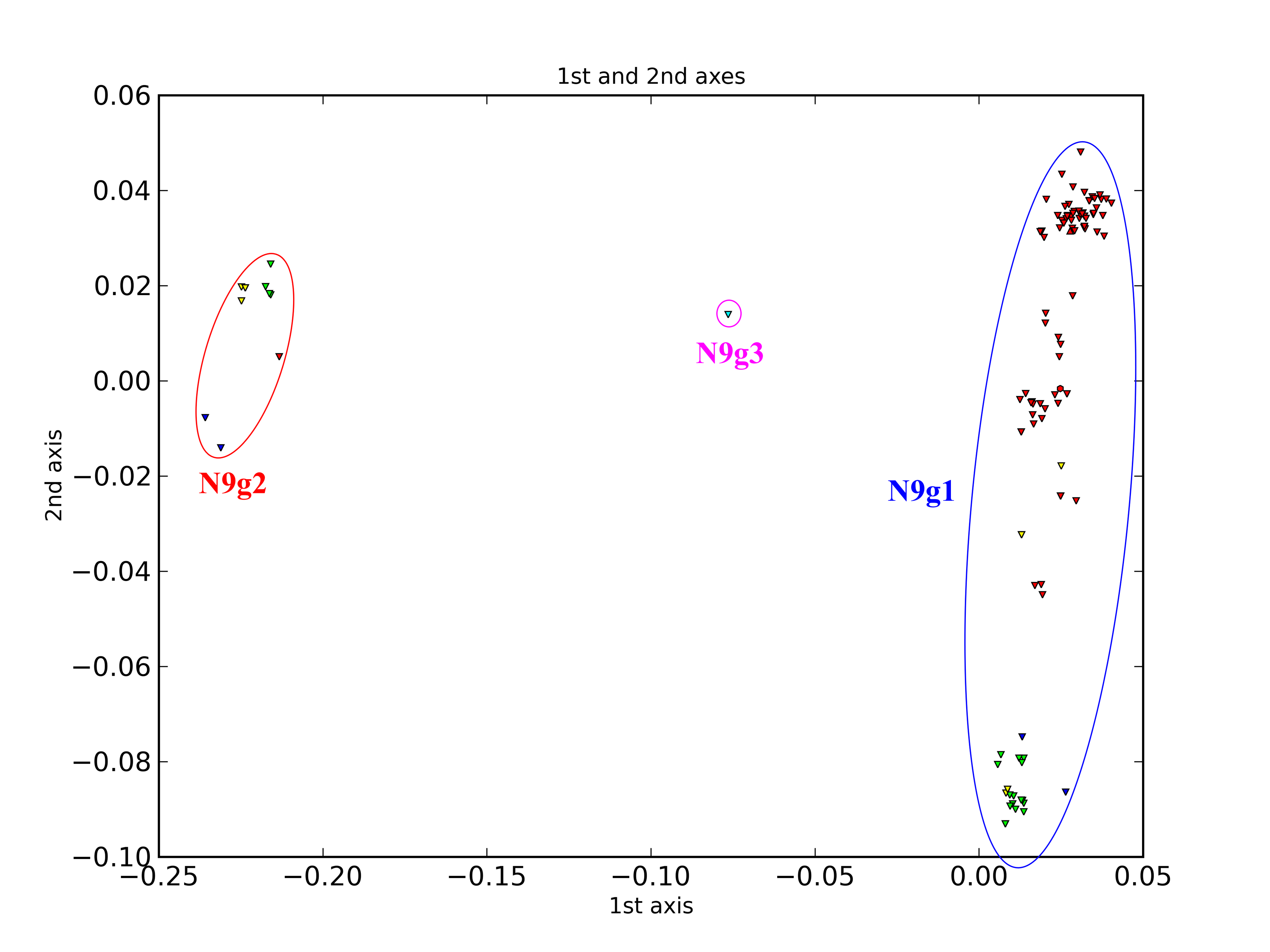

Supplement: Figure S5 — PCCORD of N9 subtype influenza viruses. The sequences are coded for host (shape of dot) and geographic origin (color) as for figure 3. (1.45 MB TIF) [file pone.0014454.s008.tif]

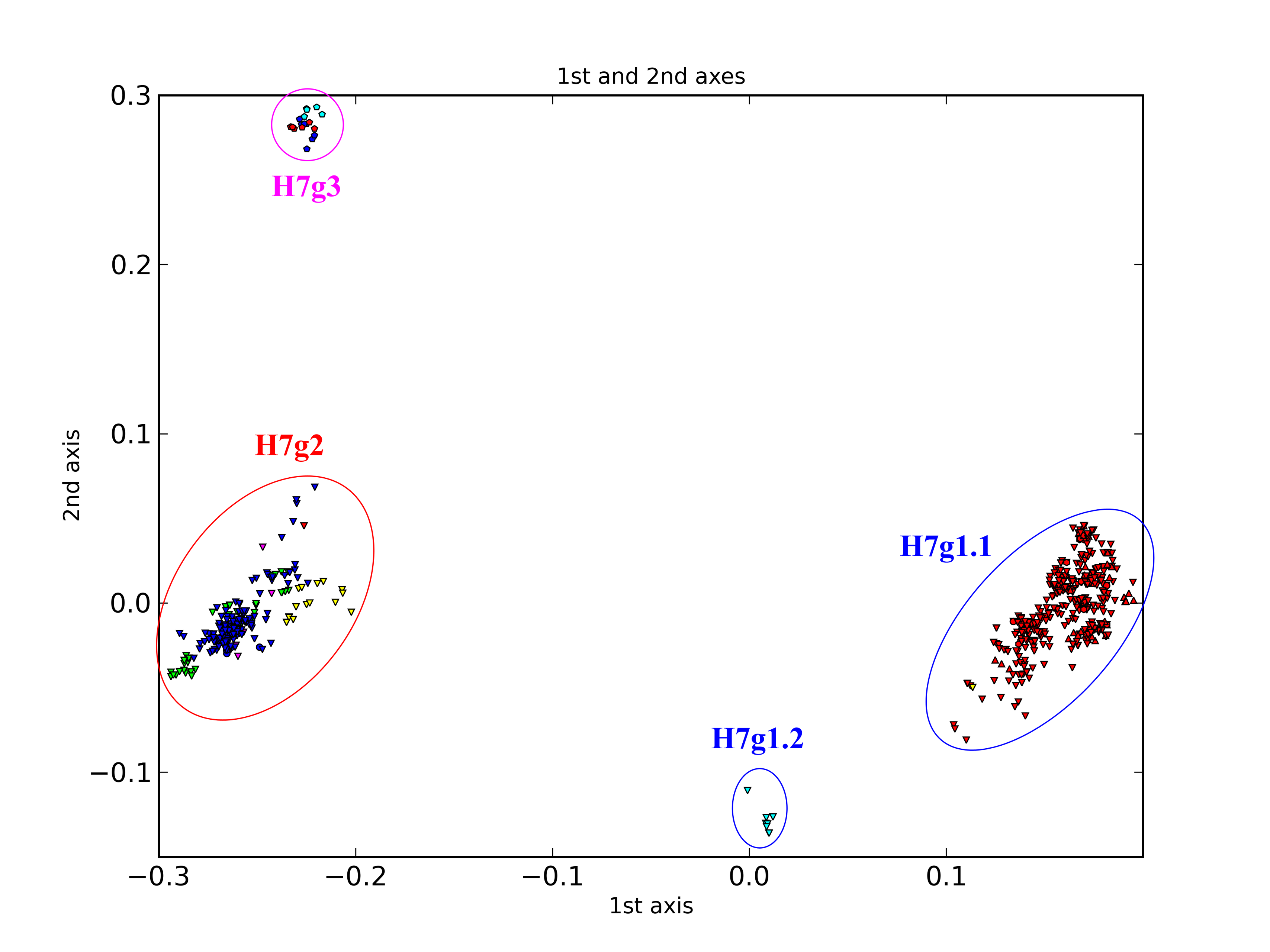

Supplement: Figure S6 — PCCORD of H7 subtype influenza viruses. The sequences are coded for host (shape of dot) and geographic origin (color) as for figure 3. (1.47 MB TIF) [file pone.0014454.s009.tif]

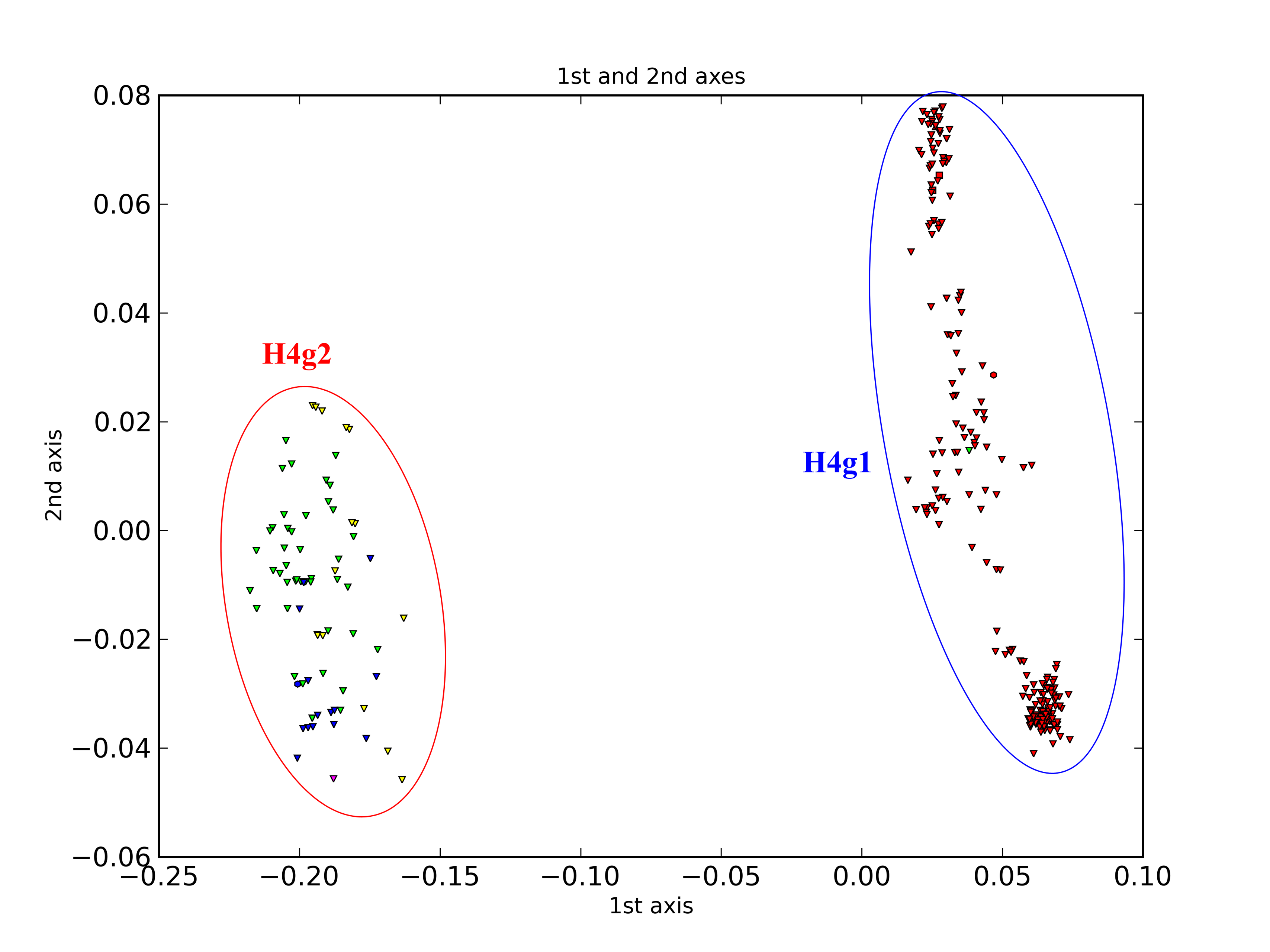

Supplement: Figure S7 — PCCORD of H4 subtype influenza viruses. The sequences are coded for host (shape of dot) and geographic origin (color) as for figure 3. (1.60 MB TIF) [file pone.0014454.s010.tif]
